# Pandora’s Box

**DOI:** 10.1192/bji.2025.10071

**Published:** 2025-11

## Recharge your brain batteries with lithium

Did you know that our brains contain lithium, and that this has a part to play in preserving our cognitive function as we age? It has been observed for many years that cognitive impairment is less likely in people receiving treatment with lithium for bipolar disorder. Several studies have demonstrated the neurotrophic and neuroprotective effect of lithium, and this was seen as a therapeutic mechanism relevant to the pathophysiology of the mood disorder. It has been suggested that the effects of lithium on the brain may be also beneficial with respect to cognitive function and prevention of dementia in people with bipolar disorder, but no convincing evidence had been found until now.

Researchers analysing a variety of metals in the brains of people with mild cognitive impairment, a prelude to Alzheimer’s dementia, identified lithium as the only metal that showed significantly reduced levels. In people with Alzheimer’s dementia, lithium brain bioavailability was reduced further by sequestration with amyloid plaques. The investigators proceeded to examine the effects of lithium on cognitive function in animals. They depleted lithium from the diets of wild-type (healthy) mice and mouse models of Alzheimer’s dementia. A 50% reduction in cortical lithium levels in the animal brains was associated with a series of changes, including increased amyloid-β levels, accumulation of phospho-tau pro-inflammatory microglial activation, and synaptic, axon and myelin loss, and these were associated with accelerated cognitive decline in the animals. When the animals were treated with lithium orotate (rather than carbonate), which has reduced potential for amyloid binding, the pathological changes and memory loss were prevented in both the ageing wild-type and Alzheimer’s dementia mouse models.

Could lithium prove to be an option for prevention and treatment of Alzheimer’s dementia in humans? Worth exploring, but let’s not forget lithium’s effects on the kidneys long term.

**Lyoo IK, Dager SR, Kim JE, Yoon SJ, Friedman SD, Dunner DL, et al**. Lithium-induced gray matter volume increase as a neural correlate of treatment response in bipolar disorder: a longitudinal brain imaging study. *Neuropsychopharmacology* 2010; **35**: 1743–50.

**Aron L, Ngian KN, Qiu C, Choi J, Liang M, Drake DM, et al.** Lithium deficiency and the onset of Alzheimer’s disease. *Nature* 2025; **645**: 712–21.

## Taking care of your hearing keeps dementia away

Poor hearing is not good for our health and quality of life in general as we grow older. Beware: as well as risking late-onset paranoid psychosis, not hearing may add to our risk of dementia. At least, so claims a recent study that examined the role of hearing in cognitive function.

The well-known Framingham Heart Study (FHS) has provided a wealth of useful data beyond cardiovascular ailments. Participants from the FHS’s original cohort (1977–1979) and offspring cohort (1995–1998) were recruited in this study, which examined prospectively whether using a hearing aid (self-reported) was associated with a reduction in the risk of dementia. Participants aged 60 years or over who had no dementia when they underwent audiometry at examination as part of the FHS in 1977–1979 (original cohort) and 1995–1998 (offspring cohort) were followed up for onset of dementia for up to 20 years.

The researchers found that 20% of the participants developed dementia (all-cause) and 42% of these were less than 70 years old at the time of the hearing assessment. Twenty-nine per cent of those with no hearing loss showed a lower risk of all-cause dementia. Among people over 70 years old, those with hearing loss who used hearing aids had a 61% lower risk for dementia than those who did not use hearing aids. However, bear in mind that some doubt was cast on the paper’s claims (see comment by Dascheiff R, Something doesn’t sound right, 1 Oct 2025).

The message from the researchers is, if we want to keep our memory, best make sure our hearing is up to scratch; if hard of hearing, keep the hearing aids handy!

**Francis L, Seshadri S, Dillard LK, Kujawa SG, Welling DB, Alcabes RL, et al.** Self-reported hearing aid use and risk of incident dementia. *JAMA Neurol* [Epub ahead of print] 18 Aug 2025. Available from: https://doi.org/10.1001/jamaneurol.2025.2713.

## Time to globalise research

Health research has been based mainly in high-income countries for a variety of reasons we can all think of and that there is no need to elaborate. However, it is time to rethink, as the world situation is rapidly changing. With human diversity and movement of people across countries, together with ongoing environmental and political changes, at a time that we are faced with increasing rates of ill-health, including neurological and psychiatric morbidities, it is imperative to take a more global approach to research and make research facilities available to all parts of the world, to the benefit of all. Pandora reported previously on findings of a genetic study in Brazil that have the potential to improve local people’s health and reduce morbidity (Pérez Oretga R. Massive DNA sequencing effort reveals how colonization shaped Brazil’s genetic diversity. *Science* 15 May 2025. Available from: https://doi.org/10.1126/science.zft36gr).

Global research is not only feasible at reasonable costs, as demonstrated by two recent large-scale field data acquisition programmes carried out in India and Tanzania, but also highly useful universally. The programmes implemented concerned the acquisition of neurophysiological (specifically, electroencephalographic) data, which can enrich our understanding of the human brain, the body organ most sensitive to both internal and external environmental stimuli. The studies generated high-throughput electroencephalographic data, which were found to be comparable with those obtained in controlled laboratory settings. This demonstrated that low- and middle-income countries have the potential to implement large-scale programmes that could help to increase our understanding of various health conditions.

It is time for researchers to think globally and aim for more collaboration between high- and low-income countries, enabling training and research to be carried out on-site in the latter, instead of the current practice of brain drain, which benefits the rich countries to the detriment of the poor.

**Vianney J-M, Swaminathan S, Newson JJ, Parameshwaran D, Subramaniyam NP, Roy SS, et al.** EEG data quality in large-scale field studies in India and Tanzania. *eNeuro* 2025; 12: 2–12.

## The phantom limb story

After limb amputation, some people continue to feel the presence of their absent limb. This phenomenon of the phantom limb was described as early as the 16th century by French military surgeon Ambroise Paré, yet we still do not fully understand the pathophysiological mechanism behind it. Some people also experience troublesome phantom limb pain. Various theories exist to explain the pain, including psychogenic factors, peripheral nerve changes at the stump site, and spinal cord-level hyperactivity to nociceptive signals, together with a decrease in inhibitory activity in supraspinal sites.

Cortical reorganisation, with neighbouring somatosensory brain areas taking over the ‘amputated region’, has been considered. However, recent research has provided evidence that the brain ‘body map’ persists after amputation. The authors tracked the evolution of cortical representations of hand and face (lips) in three adult participants before and after amputation compared with controls. They carried out functional magnetic resonance imaging before and up to 5 years after amputation and demonstrated persistence in the representation of the limb in the appropriate brain region despite its physical absence.

However, the authors did not discuss whether the study participants experienced a phantom limb or phantom limb pain. Knowing whether the body map persists even if the individual accepts the absence of the limb could help us to understand the phenomenon better. It would also be interesting to do a similar study examining the brain representation of an intact limb that the individual believes not to belong, i.e. they do not believe it to be theirs. Body integrity identity disorder or body integrity dysphoria is a very rare phenomenon that has on occasions led to limb amputation!

**Schone HR, Maimon-Mor RO, Kollamkulam M, Szymanska MA, Gerrand C, Woollard A, et al.** Stable cortical body maps before and after arm amputation. *Nat Neurosci* [Epub ahead of print] 21 Aug 2025. Available from: https://doi.org/10.1038/s41593-025-02037-7.

